# Bilateral quadriceps tendon rupture as the presenting manifestation of chronic kidney disease

**DOI:** 10.4103/0971-4065.78079

**Published:** 2011

**Authors:** N. A. Wani, H. A. Malla, T. Kosar, I. M. Dar

**Affiliations:** Department of Radiodiagnosis and Imaging, Sher-I-Kashmir Institute of Medical Sciences, Srinagar, India; 1 Department of Medicine, Sher-I-Kashmir Institute of Medical Sciences, Srinagar, India

**Keywords:** Chronic renal disease, hyperparathyroidism, magnetic resonance imaging, quadriceps tendon rupture

## Abstract

Bilateral painful knees with loss of extension in a patient with chronic kidney disease (CKD) may be due to spontaneous quadriceps tendon rupture. This rare complication is usually seen in patients on long term dialysis. We present a case of bilateral spontaneous quadriceps tendon rupture demonstrated by magnetic resonance imaging in a 20-year-old woman who on evaluation was found to have CKD.

## Introduction

Spontaneous tendon ruptures involving quadriceps, triceps, and finger tendons, characterising uremic tendonopathy, are seen in about 15% of patients with chronic kidney disease (CKD) on hemodialysis.[1] For best results, early repair is recommended, thus necessitating early diagnosis.[1,2] Development of bilateral simultaneous quadriceps tendon ruptures in CKD may correlate with the duration of renal disease and length of dialysis.[2] Most patients have secondary hyperparathyroidism.[1,2] We report a case of bilateral quadriceps tendon rupture diagnosed on magnetic resonance imaging (MRI) as the presenting manifestation of CKD.

## Case Report

A 20-year-old woman presented with pain and swelling of both knees from last 10 days that started while coming downstairs and progressively increased. She was unable to stand without support. There was no significant medical history. On examination, she was afebrile, asthenic, and pale; pulse was 80 beats/min, blood pressure was 130/80 mmHg. Both knees were swollen and tender, and revealed defects above low-lying patellae. There was no active extension possible against gravity; patellar reflex could not be elicited on either side. Laboratory investigations revealed hemoglobin of 7.5 g/dL. Urinalysis showed albuminuria (1+), urinary sediment had 3–4 red cells/high power field; there were no casts, pus cells, or sugar; 24-h urinary protein excretion was 1.5 g/d. Blood chemistry results were as follows: blood urea of 130 mg/dL, serum creatinine 6 mg/dL with estimated creatinine clearance of 19.3 mL/min; serum calcium was 9 mg/dL, phosphate was 5 mg/dL, parathyroid hormone (PTH) level was 600 pg/mL; blood pH was 7.2, bicarbonate level was 16 mEq/L, potassium was 3 mEq/L; sodium and liver function tests were normal. Serological tests did not suggest any evidence of hepatitis (B, C) or HIV infection; antinuclear antibodies and antidouble stranded antibodies were absent.

**Figure 1:**
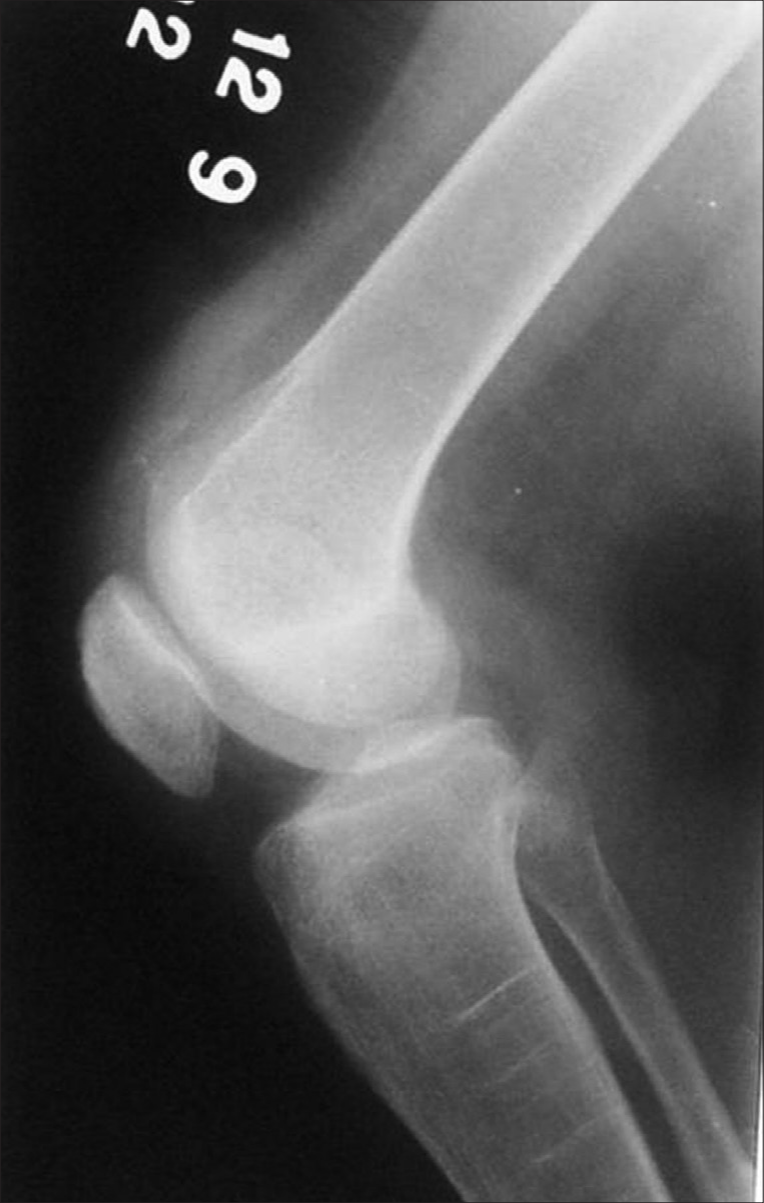
X-ray of right knee lateral view shows a slight inferior displacement of patella with loss of quadriceps tendon bulge just above it; calcification is seen in suprapatellar region

**Figure 2:**
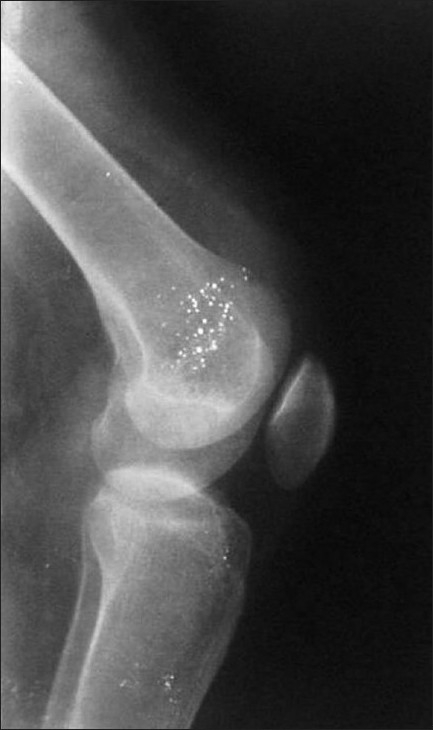
X-ray of lateral view of left knee shows inferior displacement of patella with loss of suprapatellar quadriceps bulk

**Figure 3:**
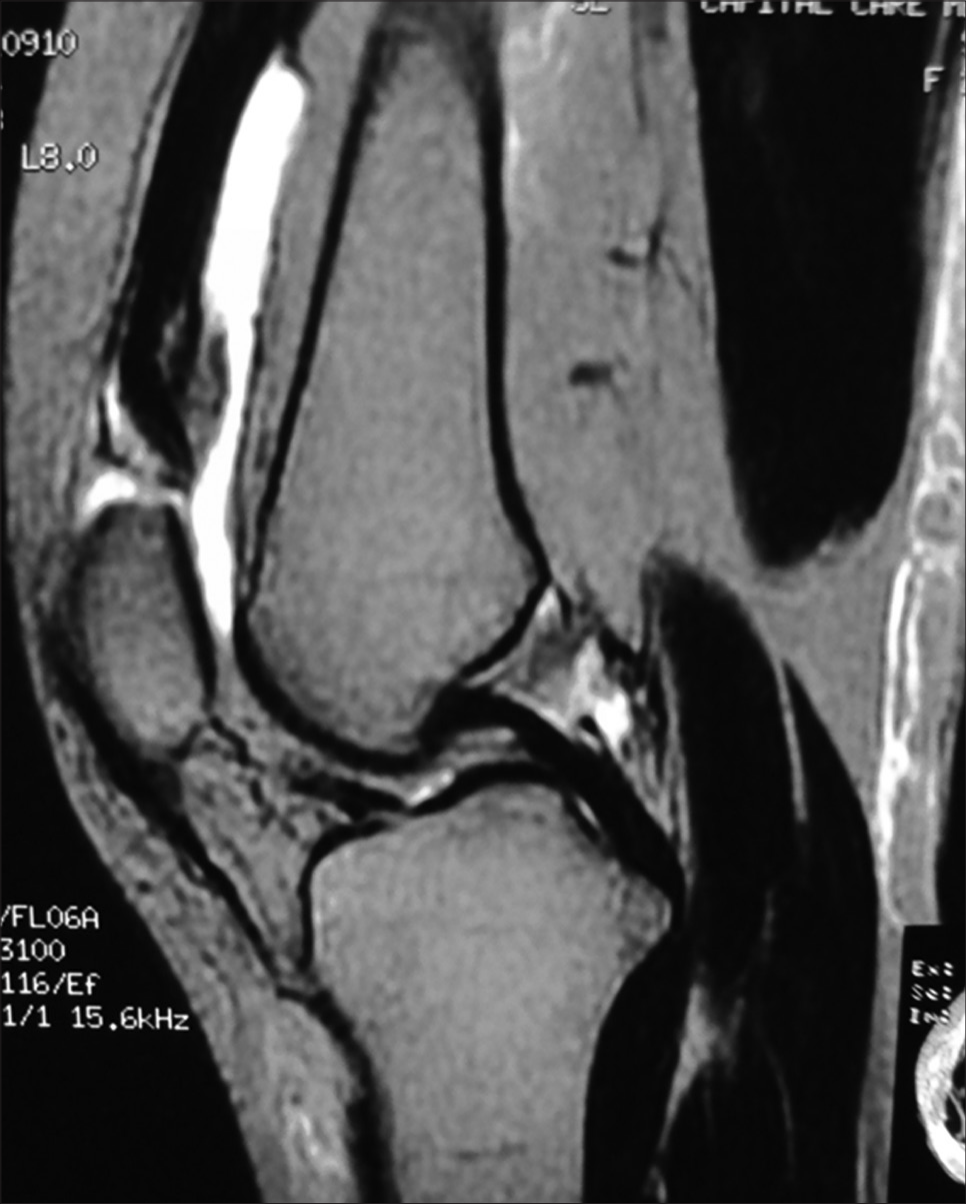
T2-weighted sagittal MR image of right knee showing discontinuity between lower end of hypointense signal intensity quadriceps tendon and upper pole of patella with intervening hyperintense signal intensity; joint effusion is seen

**Figure 4:**
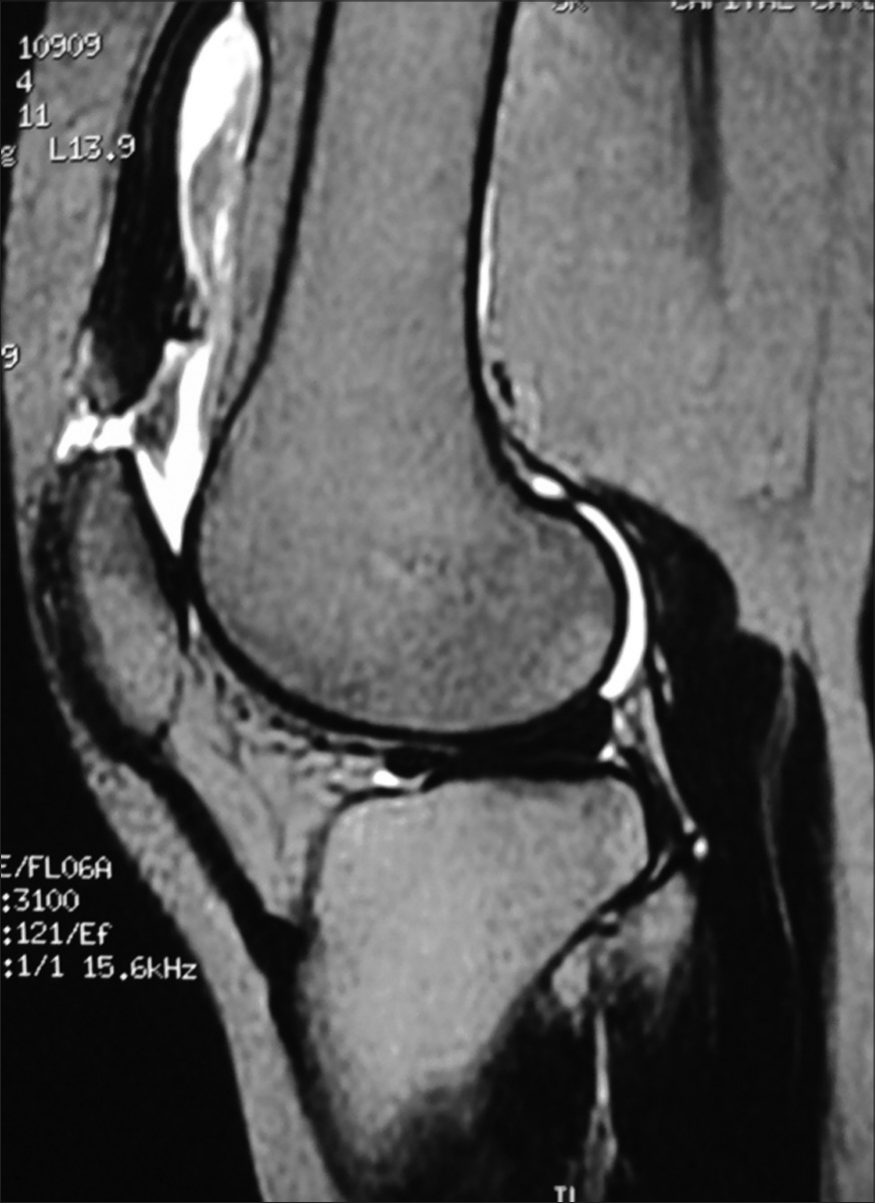
T2-weighted sagittal MR image of left knee joint shows discontinuity between quadriceps tendon and upper pole of patella filled with hyperintense signal intensity

X-ray of knees showed slightly low-lying patella with diminished bulk of quadriceps tendon just above the patella in either knee [Figures 1 and 2]; soft tissue calcification was seen in the suprapatellar region of the right knee [Figure 1]. T2-weighted MRIs in sagittal plane revealed discontinuity between distal quadriceps tendon and upper pole of patella in both knee joints with the intervening space filled with hyperintense signal intensity, in definite contrast to hypointense signal intensity of tendon [Figures 3 and 4]; small joint effusion was seen in the suprapatellar region on either side [Figures 3 and 4]. Bones, menisci, and cruciate ligaments were normal. A diagnosis of bilateral spontaneous quadriceps tendon rupture with stage IV CKD with hyperparathyroidism and metabolic acidosis was made.

Patient was treated with intravenous fluids, sodium bicarbonate, and potassium chloride judiciously, and monitored carefully for the correction of acidosis and hypokalemia. Immediate cause of hypokalemia could not be ascertained; however, the potassium levels remained normal after initial correction. Ultrasound showed bilateral small and echogenic kidneys with loss of corticomedullary differentiation. Renal biopsy revealed advanced glomerulosclerosis. After stabilization, surgical repair of the quadriceps tendons was performed by fixing the tendon to the patella through nonabsorbable sutures. Knees were splinted in extension followed by gradual weight bearing after 4 weeks and complete weight bearing at 7–8 weeks. She gradually gained the power and movements in her knees and could stand independently after 4 months. Peritoneal dialysis was continued and her PTH level came down with continued vitamin D therapy.

## Discussion

Quadriceps tendon is an important component of extensor mechanism of knee joint and is formed by three vasti and rectus femoris muscles just above patella. Quadriceps tendon ruptures are uncommon; resulting from trauma in the elderly. Quadriceps tendon ruptures usually during a rapid, eccentric contraction of the quadriceps muscle, with planted foot and partially flexed knee, due to fall or direct blow.[3] Bilateral ruptures are very rare and strongly associated with systemic illnesses like hyperparathyroidism, renal failure, rheumatoid arthritis, gout, obesity, systemic lupus erythrematosus steroid use, and diabetes mellitus. Usual site of rupture is 0–2 cm from upper pole of patella through a weakened and degenerated segment of tendon, as a result of underlying systemic disease.

Weakness and rupture of tendons in CKD has been shown to be correlated with the duration of renal failure and length of treatment with hemodialysis. The resulting malnutrition, ß_2_-amyloidosis, and accumulation of uremic toxins – all classical complications of long-term chronic hemodialysis – have been suggested to be causative factors for spontaneous tendon rupture. The unknown and untreated nature of renal failure in our case along with elevated serum PTH and low pH stresses the role of hyperparathyroidism and metabolic acidosis, the classical complications of renal failure, in the pathogenesis of uremic tendonopathy. Metabolic acidosis may result in tendon degeneration as a result of disruption of the structure of protein-polysaccharide complex, which is responsible for the maturation of collagen.[4,5] Hyperparathyroidism in renal failure is the result of retention of phosphate due to decreased glomerular filtration rate, with consequent hypocalcemia resulting in parathyroid gland stimulation.[6] Restoration of serum calcium and phosphate is at the expense of increased bone resorption due to high PTH. Secondary hyperparathyroidism causes dystrophic calcification and subperiosteal bone resorption, which respectively weaken the tendon and osteotendinous junction.[6,7] Relatively minor trauma can then cause spontaneous rupture of tendon at tendon–bone junction.[5–7]

After the quadriceps tendon rupture, patient reports pain and loss of function of the knee joint. Examination reveals swelling around knee, suprapatellar gap, and loss of extension at knee joint. Pain and swelling make clinical diagnosis inconclusive and hence need for imaging. Imaging is the key for proper management of quadriceps tendon rupture, and optimal outcome in a complete rupture is possible only after early diagnosis and timely repair.[8] X-ray shows low-lying patella, dystrophic calcification, and indistinct bulk of quadriceps tendon; underlying changes of hyperparathyroidism, like bone resorption may also be apparent. Ultrasonography is a reliable and relatively cheap modality for diagnosing tendon rupture; however it is operator dependent.[5] MRI is the imaging modality of choice for the derangements of knee joint including its extensor apparatus. MRI localizes the site of tendon disruption and determines the extent of rupture as either partial or complete. On MRI, tendon is seen as hypointense structure on T2-weighted sequences due to sparsity of free protons. Rupture is seen as an area of disruption in the continuity of the hypointense tendon filled with hemorrhage and edema which is seen as hyperintense signal intensity on T2-weighted and as hypointense signal on T1-weighted images.[8,9] Bone edema may be seen in the patella around tendon insertion; patella is displaced inferiorly. Quadriceps tendon may appear taut and retracted, and patellar tendon may show slackening. Besides tendon tear, MRI may show tear of menisci, cruciate, and collateral ligaments, and fluid/blood within knee joint. MRI is also useful in the follow-up after surgical repair of the ruptured tendon.[9]

Treatment of complete rupture is surgical repair with sutures fixing the tendon with the patella.[5,10] Partial tears of the tendon may be treated conservatively; repair may be performed if it fails. Following surgery, knee joint is immobilized for 4–6 weeks followed by graded weight bearing.[9] Underlying hyperparathyroidism needs to be addressed to prevent further tendon ruptures. Calcium and vitamin D analogues are given; total parathyroidectomy may be performed with autotransplantation of a parathyroid gland to avoid hypoparathyroidism.[6]

## Conclusion

Bilateral spontaneous quadriceps tendon rupture, on MRI of painful swollen knees, may be the initial manifestation of CKD complicated by hyperparathyroidism. MRI optimizes diagnosis and management of quadriceps rupture. Once such a rupture is identified at MRI, CKD with hyperparathyroidism needs to be excluded.
